# The T-Type Calcium Channel Cav3.2 in Somatostatin Interneurons in Spinal Dorsal Horn Participates in Mechanosensation and Mechanical Allodynia in Mice

**DOI:** 10.3389/fncel.2022.875726

**Published:** 2022-04-08

**Authors:** Yu-Ru Zhi, Feng Cao, Xiao-Jing Su, Shu-Wen Gao, Hao-Nan Zheng, Jin-Yan Jiang, Li Su, Jiao Liu, Yun Wang, Yan Zhang, Ying Zhang

**Affiliations:** ^1^Neuroscience Research Institute, Department of Neurobiology, School of Basic Medical Sciences, Key Laboratory for Neuroscience, Ministry of Education/National Health Commission of China, Peking University, Beijing, China; ^2^Stroke Center and Department of Neurology, The First Affiliated Hospital of USTC, Division of Life Sciences and Medicine, University of Science and Technology of China, Hefei, China; ^3^Department of Gastroenterology, Peking University First Hospital, Beijing, China; ^4^Center of Medical and Health Analysis, Peking University Health Science Center, Beijing, China; ^5^PKU-IDG/McGovern Institute for Brain Research, Peking University, Beijing, China

**Keywords:** intraspinal injection, knockdown, low-voltage activated calcium channel, SOM neurons, spinal cord slice recording

## Abstract

Somatostatin-positive (SOM^+^) neurons have been proposed as one of the key populations of excitatory interneurons in the spinal dorsal horn involved in mechanical pain. However, the molecular mechanism for their role in pain modulation remains unknown. Here, we showed that the T-type calcium channel Cav3.2 was highly expressed in spinal SOM^+^ interneurons. Colocalization of *Cacna1h* (which codes for Cav3.2) and SOM*^tdTomato^* was observed in the *in situ* hybridization studies. Fluorescence-activated cell sorting of SOM*^tdTomato^* cells in spinal dorsal horn also proved a high expression of *Cacna1h* in SOM^+^ neurons. Behaviorally, virus-mediated knockdown of *Cacna1h* in spinal SOM^+^ neurons reduced the sensitivity to light touch and responsiveness to noxious mechanical stimuli in naïve mice. Furthermore, knockdown of *Cacna1h* in spinal SOM^+^ neurons attenuated thermal hyperalgesia and dynamic allodynia in the complete Freund’s adjuvant-induced inflammatory pain model, and reduced both dynamic and static allodynia in a neuropathic pain model of spared nerve injury. Mechanistically, a decrease in the percentage of neurons with Aβ-eEPSCs and Aβ-eAPs in superficial dorsal horn was observed after *Cacna1h* knockdown in spinal SOM^+^ neurons. Altogether, our results proved a crucial role of Cav3.2 in spinal SOM^+^ neurons in mechanosensation under basal conditions and in mechanical allodynia under pathological pain conditions. This work reveals a molecular basis for SOM^+^ neurons in transmitting mechanical pain and shows a functional role of Cav3.2 in tactile and pain processing at the level of spinal cord in addition to its well-established peripheral role.

## Introduction

Neuropathic pain arising from a lesion or disease of the somatosensory system, including peripheral fibers (Aβ, Aδ and C fibers) and central neurons, affects 7–10% of the general population (Colloca et al., 2017). These patients often experience spontaneous and stimulus-evoked pain (allodynia and hyperalgesia). Distinct from inflammatory pain or cancer pain, antidepressants and anticonvulsants are recommended as the first-line pharmacological treatment options for neuropathic pain (Wright and Rizzolo, 2017). However, pharmacological treatments for neuropathic pain are effective in < 50% of patients (Finnerup et al., 2015). Novel therapeutic agents for neuropathic pain are needed.

Mechanical allodynia, in which normally non-painful mechanical stimuli become painful, is a highly prevalent condition in patients with neuropathic pain (Bouhassira et al., 2005). The spinal dorsal horn, the major integration center of peripheral sensory information, is deemed the key site that gives rise to mechanical allodynia (Peirs and Seal, 2016; Todd, 2017; Moehring et al., 2018). Normally, tactile afferent inputs (mainly Aβ inputs) to pain-transmitting neurons in the superficial dorsal horn are gated by inhibitory neurons in the spinal dorsal horn (Coull et al., 2003; Lu et al., 2013; Petitjean et al., 2015; Cui et al., 2016; Boyle et al., 2019), as suggested by gate-control theory. However, the gate no longer works due to sensitization of the excitatory neurons or diminished inhibitory tone after nerve lesion or spinal cord injury, leading to mechanical allodynia.

Somatostatin-positive (SOM^+^) and dynorphin-positive (Dyn^+^) interneurons in the spinal cord have been demonstrated to represent pain-transmitting neurons and relevant inhibitory neurons, respectively (Duan et al., 2014). Ablation of spinal SOM^+^ neurons resulted in nearly complete loss of mechanical pain. Therefore, elucidating the unique genetic features of these neurons will enable selective targeting of these neurons for pain relief and development of novel analgesic agents. Recent RNA sequencing of SOM^+^ neurons uncovered more than 900 genes with at least twofold enrichment, among which *Cacna1h* (which codes for Cav3.2), a subtype of T-type Ca^2+^ channels, was listed as a candidate “pain gene” enriched in SOM^+^ neurons (Chamessian et al., 2018).

Cav3.2, together with Cav3.1 (*Cacna1g*), and Cav3.3 (*Cacna1i*), constitute low-voltage-activated T-type Ca^2+^ channels, which are activated at voltages near the resting membrane potential. T-type Ca^2+^ channels have been suggested to be involved in rebound depolarization (Molineux et al., 2006), burst firing (Matthews and Dickenson, 2001; Cain and Snutch, 2013), spontaneous firing (Li et al., 2017; Lauzadis et al., 2020), and subthreshold membrane potential oscillations (Crunelli et al., 2005; Choi et al., 2010). Cav3.2 is the major subtype of T-type Ca^2+^ channels in peripheral sensory neurons (Bourinet et al., 2005; Rose et al., 2013) and has been demonstrated to be primarily expressed in low-threshold mechanoreceptors (LTMRs), including Aδ- and C-LTMRs (Francois et al., 2015; Bernal Sierra et al., 2017). Moreover, C-LTMR-specific knockout revealed that Cav3.2 regulates acute mechanosensory processing and is essential for the allodynia symptoms of neuropathic pain (Francois et al., 2015). Recently, the role of Cav3.2 in the electrophysiological properties of spinal dorsal horn neurons has been revealed (Candelas et al., 2019). Selective deletion of spinal Cav3.2 blunted the transient firing patterns and rebound depolarizations and remodeled the kinetics of the action potentials in laminae II neurons. However, the functional role of Cav3.2 in spinal dorsal horn neurons remains unknown.

To elucidate the potential roles of spinal Cav3.2 in pain modulation, we investigated the localization of Cav3.2 in spinal SOM^+^ neurons using *in situ* hybridization in the current work. Furthermore, we determined the behavioral effects of selective knockdown of *Cacna1h* in SOM^+^ neurons through intraspinal injection of adeno-associated virus under normal and pathological conditions. The relevant electrophysiological mechanisms were investigated as well. In summary, our findings indicated a crucial role of Cav3.2 in spinal SOM^+^ interneurons in mechanical pain.

## Materials and Methods

### Animals

The mice used in this work, *somatostatin-IRES-Cre* (*SOM^Cre^*), *preprodynorphin-IRES-Cre* (*Pdyn^Cre^*), ai14 *ROSA26^CAG–loxP–STOP–loxP–tdTomato^* reporter, and *Cacna1h*^–/–^, were obtained from the Jackson laboratory. The wild type C57BL/6 mice were purchased from the Animal Center of Peking University Health Science Center. Littermate mice were randomly divided into control and experimental groups.

Adult male mice were used in the experiments. Mice were kept on a 12:12-h light-dark cycle (lights on at 8 AM and lights off at 8 PM) with *ad libitum* access to food and water.

### *In situ* Hybridization

Section *in situ* hybridization was performed as described in the literature (Ma et al., 1998). The *in situ* probe for *Cacna1h* (0.7 kb) was amplified using gene-specific sets of PCR primers and cDNA templates prepared from adult mouse DRG (dorsal root ganglion).

For *in situ* hybridization/tdTomato double staining, the tdTomato fluorescent signal was first imaged, followed by *in situ* hybridization. The *in situ* signals were imaged under transluminescent light and converted into pseudogreen fluorescent color. Finally, the images of *in situ* hybridization and tdTomato were merged using Photoshop software.

### Dissection of Spinal Dorsal Horn Neurons and Cell Sorting

Mice were deeply anesthetized using sodium pentobarbital (100 mg/kg). Then, the spinal cord was isolated in precooled PBS. The dorsal horn was cut under a stereomicroscope and digested in papain (5 U/ml, Cat# P4762; Sigma-Aldrich, St Louis, MO, United States) for 30 min in a CO_2_ incubator at 37°C. Next, samples were washed with ice-cold HBSS (Cat#14025092; Invitrogen, Carlsbad, CA, United States) plus HEPES (Cat#15630080; Invitrogen, Carlsbad, CA, United States) solution, followed by gentle trituration using a fire-polished pipette tip. Then, the supernatant was collected and filtered through a 40 μm cell strainer. The dissociated cells were centrifuged at 1,000 *g* for 5 min at 4°C and resuspended in Neurobasal medium (Cat# 10888022; Invitrogen, Carlsbad, CA, United States). Finally, cells were counted using a hemocytometer, and the density of the cell suspension was adjusted to 10^6^–10^7^/ml.

SOM*^tdTomato^* cells were sorted using FACS ARIA SORP (BD Biosciences, Franklin Lakes, NJ, United States) by red fluorescence intensity (excitation = 581 nm, emission = 610 nm). Captured samples were placed on ice and immediately processed for RNA isolation.

### Quantitative Real-Time Polymerase Chain Reaction

Total RNA was extracted and purified using the EASYspin Kit (Aidlab, Beijing, China). RNA concentration and purity were measured using a NanoDrop 2000c spectrophotometer (Thermo Scientific, Waltham, MA, United States). SuperScript III reverse transcriptase (Invitrogen, Carlsbad, CA, United States) was used for reverse transcription of RNA into cDNA.

Quantitative real-time polymerase chain reaction (qPCR) was performed using an ABI 7500 instrument (Applied Biosystems, Foster City, CA, United States). SYBR Green 2 × PCR Master Mix (Toyobo, Osaka, Japan) was used for PCR. The primers were designed with SnapGene software (San Diego, CA, United States) and sequences of the primers for *Cacna1h, Sst* and *Gapdh* were as follows: forward, CGGCCCTACTACGCAGACTA; reverse, GGCCTCAAAGACGAAGACGA; forward, GCTGAG CAGGACGAGATGAG; reverse, AGAAGTTCTTGCAGCCAG CT; and forward, GGTGCTGAGTATGTCGTGGA; reverse, CCTTCCACAATGCCAAAGTT. The reaction conditions were set as follows: incubation at 95°C for 1 min, 40 cycles of 95°C for 15 s, 60°C for 15 s, and 72°C for 30 s. Last, melting curve analysis was performed at 95°C for 15 s, 60°C for 1 min and 95°C for 15 s. Ct values were defined as the number of PCR cycles at which the fluorescence signals were detected. The relative expression levels of the target genes were calculated using the 2^–ΔΔCt^ method and were normalized to *Gapdh*. ΔΔCT = (Ct*Cacna1h* - Ct*_*Gapdh*_*)SOM^+^ - (Ct*Cacna1h* - Ct*_*Gapdh*_*)SOM^–^.

### Viral Vector

The AAV9-shRNA vector against mouse *Cacna1h* (NM_021415.4) was custom-made based on miR30 by Vigene Biosciences (Shandong, China). The *Cacna1h*-shRNA and non-silence shRNA sequence were as follows: TCTGAGTCTGTGCACAGTATCTA and CAGGCAGAAGTA TGCAAAGCAT.

### Intraspinal Stereotaxic Injection

Mice were anesthetized using isoflurane (3% during induction, 2–3% during maintenance) and mounted on a stereotaxic apparatus using the mouse spinal adaptor (RWD Life Science, Shenzhen, China). The skin at the middle/lower back was incised about 2 cm along the midline. By removing the muscles and ligaments, the surface of the lumbar spinal cord was exposed. The pipette was centered above the posterior median sulcus. Next, the tip was moved 500 μm laterally and lowered 200–300 μm into the spinal cord. Then, 0.3 μl of virus was injected at a rate of 50 nl/min. At the end of the injection, at least 5 min was allowed before slowly retracting the pipette. Finally, the skin was closed, and the mouse was returned to its home cage.

### Establishment of Chronic Pain Models

For the inflammatory pain model, 10 μl complete Freund’s adjuvant (CFA, Cat# F5881; Sigma-Aldrich, St Louis, MO, United States) was injected into the plantar surface of the left hind paw. Behavioral tests were performed at the indicated time points.

For the neuropathic pain model, surgery for spared nerve injury (SNI) was performed. After anesthetization using isoflurane, the sciatic nerve near the thigh region was exposed. Then, the common peroneal nerve and tibial nerve were ligated and cut while leaving the sural nerve intact. Sham controls underwent exposure of the thigh region without any damage to the nerve.

### Behavioral Tests

The behavioral tests were performed in a double-blind manner by two experimenters. One experimenter was responsible for grouping and numbering the rats. The other who performed the behavioral tests was unaware of the grouping of the whole experiment.

#### Open Field Test

The open field apparatus consisted of a clear Plexiglas box (50 cm × 50 cm × 40 cm). The mouse was gently placed in the center of the arena and was allowed to explore the area in a room with dim light for 30 min. The movement of mouse was recorded using a digital camera above the arena and movement traces were analyzed with Smart V3.0 (Panlab, Spain) software.

#### Rotarod Test

Mice were tested on an accelerating rotarod (IITC, United States, Woodland Hills, CA, United States). During the training session, the mouse was placed on a rotarod continuously moving at 5 rpm for 1 min. If the mouse fell, it was placed back on the rotarod, and the 1 min trial was started again. Training occurred on two consecutive days. On the testing day, the rotarod began at 4 rpm and accelerated to 40 rpm over 5 min. The latency to fall was automatically recorded. The experiment was repeated twice with an interval of 20 min, and the average value was used.

#### Brush Test and Dynamic Allodynia Assay

Mice were placed separately in plexiglass chambers with an elevated mesh floor and habituated for 15–20 min. Then, the plantar surface of the hind paw was stimulated by light stroking using a fine paintbrush from the heel to the toe. No evoked movement was scored as 0, and walking away or occasionally brief paw lifting (∼1 s or less) was scored as 1. The test was repeated 3 times with an interval of 5 min, and the average score was used.

For the dynamic allodynia assay, a similar stimulation was applied, but the following scales were used: 0, walking away or occasionally brief paw lifting (∼1 s or less); 1, sustained lifting (more than 2 s) of the stimulated paw toward the body; 2, strong lateral lifting above the level of the body; and 3, flinching or licking of the affected paw (Duan et al., 2014).

#### Sticky Tape Test

A circular adhesive tape of 1 cm in diameter was stuck onto the hind paw plantar surface of the mouse. The latency to bite or lick to remove the tape was recorded.

#### Pinprick Test

The plantar surface of the hind paw was stimulated using a pin without skin penetration. The number of withdrawal responses per 10 trials with an interval of 1 min was recorded and the percentage of response was calculated.

#### Pinch Test

Mice were placed separately in plexiglass chambers on an elevated glass floor. An alligator clip was applied to the plantar surface of the hind paw between the footpad and the heel. Then, the mice were placed back into the chamber and the licking latency was recorded. A cutoff time of 60 s was set to avoid tissue injury.

#### von Frey Filament Test

Mice were placed separately in plexiglass chambers with an elevated mesh floor and habituated for 15–20 min. A set of calibrated von Frey filaments (0.008–6 g) was used to stimulate the plantar surface of the hind paw. The 50% paw withdrawal threshold was determined using Dixon’s up-down method (Chaplan et al., 1994).

#### Acetone Test

The acetone evaporation assay was performed as reported previously (Dhandapani and Karthigeyan, 2018). Mice were placed separately in plexiglass chambers with an elevated mesh floor and habituated for 15–20 min. A small drop of acetone was deposited on the hind paw. Behaviors were scored according to the following scales: 0, no response; 1, brief lift, sniff, flick, or startle; 2, jumping, paw shaking; 3, multiple lifts, paw lick; 4, prolonged paw lifting, licking, shaking, or jumping; and 5, paw guarding. The test was repeated three times with an interval of 5 min, and the average score was used.

#### Hargreaves Test

Mice were placed separately in plexiglass chambers on an elevated glass floor. A beam of radiant heat was applied onto the plantar surface of the hind paw. The latency to withdraw the hind paw was measured. Beam intensity was adjusted appropriately, so that naïve mice displayed a latency of approximately 15 s. A cutoff time of 30 s was used to avoid tissue damage. The test was repeated three times with an interval of 10 min, and the average value was used.

#### Hot Plate Test

Before testing, mice were placed on a hot plate apparatus (BiosebLab, Pinellas Park, FL, United States) to acclimate to the environment for 15 min. During the test, the hot plate was set at 54°C, and the latency to hind paw licking was recorded. To avoid tissue injury, a cut-off time of 30 s was set. The test was repeated three times with an interval of 10 min, and the average value was used.

### Spinal Cord Slice Electrophysiology

#### Spinal Cord Slice Preparation

Parasagittal spinal cord slices with DRG and dorsal roots attached (10–20 mm) were prepared as previous studies (Zhang et al., 2018). Mice (12–14 weeks) were anesthetized and perfused intracardially with 4°C NMDG substituted artificial cerebrospinal fluid (NMDG-ACSF) containing (in mM) 93 *N*-methyl-D-glucamine (NMDG), 2.5 KCl, 1.2 NaH_2_PO_4_, 30 NaHCO_3_, 20 HEPES, 25 glucose, 2 thiourea, 5 Na-ascorbate, 3 Na-pyruvate, 0.5 CaCl_2_, 10 MgSO_4_ and 3 glutathione (GSH). The osmolarity was 310–320 mOsm and pH was titrated to 7.3–7.4 with HCl. The lumbar spinal cord was quickly removed to ice-cold oxygenated NMDG-ACSF and the spinal cord with full-length dorsal root and DRG attached was cut on a vibratome (VT1200S, Leica, Germany). The slices were then incubated in NMDG-ACSF for 10 min at 32°C, followed by *N*-2-hydroxyethylpiperazine-*N*-2-ethanesulfonic acid (HEPES) ACSF containing (in mM) 92 NaCl, 2.5 KCl, 1.2 NaH_2_PO_4_, 30 NaHCO_3_, 20 HEPES, 25 glucose, 2 thiourea, 5 Na-ascorbate, 3 Na-pyruvate, 2 CaCl_2_, 2 MgSO_4_ and 3 GSH (pH 7.3–7.4, 310–320 mOsm, oxygenated with 95% O_2_ and 5% CO_2_) for additional 1 h at 25°C. Slices were then transferred to a heating recording chamber and perfused with recording ACSF at 6–8 ml/min at 32°C.

#### Patch-Clamp Recordings and Dorsal Root Stimulation

Whole-cell recording experiments were performed as described previously (Zhang et al., 2018). Recordings were made from randomly picked neurons in the laminae I-II_*o*_ using oxygenated recording ACSF containing (in mM) 125 NaCl, 2.5 KCl, 2 CaCl_2_, 1 MgCl_2_, 1.25 NaH_2_PO_4_, 26 NaHCO_3_, 25 D-glucose, 1.3 sodium ascorbate and 3.0 sodium pyruvate, with pH at 7.3 and measured osmolality at 310–320 mOsm. The internal solution contains (in mM): potassium gluconate 130, KCl 5, Na_2_ATP 4, NaGTP 0.5, HEPES 20, EGTA 0.5, pH 7.28 with KOH, and osmolality at 310–320 mOsm. Data were acquired with pClamp 10.0 software using MultiClamp 700B patch-clamp amplifier (Molecular Devices, CA, United States) and Digidata 1550B (Molecular Devices, CA, United States). Responses were low-pass filtered on-line at 2 kHz and digitized at 5 kHz.

Dorsal root stimulation was conducted by a bipolar suction electrode (A-M Systems, WA, United States) connected with an ISO-Flex stimulus isolator (AMPI, Jerusalem, Israel). Stimulation at 25 μA (pulse widths 0.1 ms), 0.05 Hz was used to screen Aβ fiber-mediated synaptic responses in the dorsal horn. To record Aβ-eEPSCs, membrane potential was held at −70 mV to minimize evoked inhibitory postsynaptic currents (eIPSCs). To facilitate eIPSCs recording, the membrane potential was held at −45 mV. To record dorsal root stimulation-evoked EPSP/APs (Aβ-eEPSP/eAPs), current-clamp recordings were performed at the resting membrane potential; for neurons with spontaneous firing, hyperpolarizing current was injected to unmask synaptic responses. The recordings were performed either under the normal ACSF or under the disinhibition condition by adding bicuculline (10 μM, Cat# 14343; Sigma-Aldrich, St Louis, MO, United States) and strychnine (2 μM, Cat# S8753; Sigma-Aldrich, St Louis, MO, United States) to ACSF.

To test the influence of *Cacna1h* knockdown in spinal SOM^+^ neurons on Aβ-eEPSCs and Aβ-eEPSP/eAPs, *Cacna1h*-shRNA or non-silence shRNA was injected within L3-L5 spinal cord segments in *SOM^Cre^* mice. SNI was performed 4 weeks after injection and mice were sacrificed for analysis at 14 days post-SNI.

### Statistical Analysis

All data are represented as the mean ± SEM. Comparisons between two groups were performed using Student’s unpaired or paired *t*-test. Comparisons between two groups at different time points were performed using two-way ANOVA with Sidak’s multiple comparisons test. Comparisons of the neuronal percentage in electrophysiological recording were performed using Chi-square test. The criterion for statistical significance was *P* < 0.05, and differences were calculated using GraphPad Prism 8.0 (San Diego, CA, United States).

## Results

### Cellular Localization of *Cacna1h* in the Spinal Dorsal Horn

**FIGURE 1:**
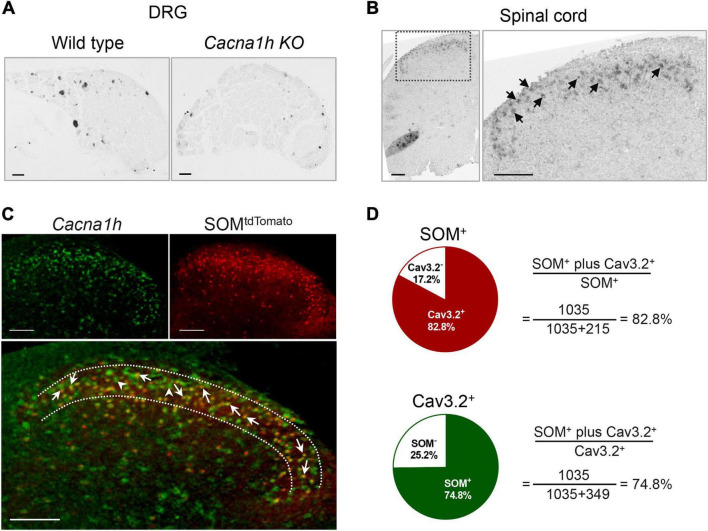
*In situ* hybridization staining of *Cacna1h* in DRG and spinal dorsal horn. **(A)** *In situ* hybridization staining of *Cacna1h* in the DRG of wild type (WT, left) and *Cacna1h* knockout (KO, right) mice. **(B)** *In situ* hybridization staining of *Cacna1h* in the lumbar segment of the spinal cord in mice. Arrows indicate *Cacna1h*^+^ cells. **(C)** *In situ* hybridization staining of *Cacna1h* (left) and SOM*^tdTomato^* neurons (right) in the spinal dorsal horn. Superimposition of the images is shown below. Arrows indicate *Cacna1h* and SOM^tdTomato^ double-positive cells, and arrowheads indicate *Cacna1h*-positive cells. **(D)** Quantification analysis of the proportion of double-positive cells in SOM^+^ (top) and Cav3.2^+^ (below) cells. Sixteen hemisections of the spinal cord from 4 male mice in each group were quantified. Scale bar, 100 μm.

As we did not find efficient anti-Cav3.2 antibody for immunostaining, we used *in situ* hybridization to observe the localization of *Cacna1h* in the spinal dorsal horn. The specificity of the probe for *Cacna1h* was confirmed by *in situ* hybridization staining of mouse DRG sections (Figure 1A, left). Intense staining of *Cacna1h* was observed in medium-diameter DRG neurons, which might be classified as Aδ-LTMRs, as previously reported (Francois et al., 2015). Moderate staining was observed in the small-diameter neurons, which might be attributed to C-LTMRs. In contrast, no remarkable positive signals were observed in DRG sections from *Cacna1h* knockout mice (Figure 1A, right).

**FIGURE 2:**
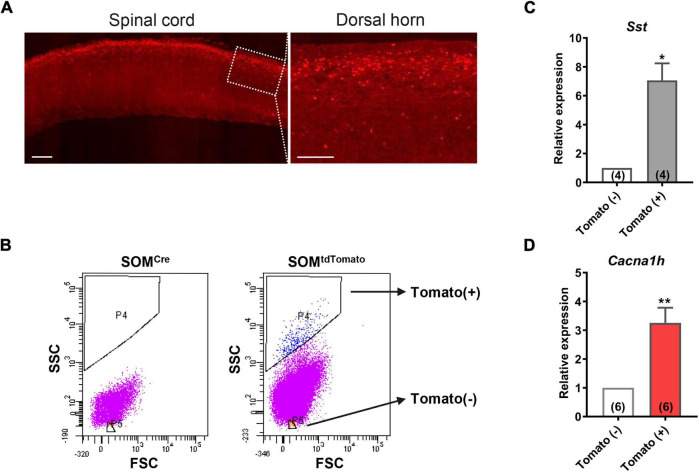
High expression of *Cacna1h* in SOM^+^ neurons in spinal dorsal horn. **(A)** Representative image of SOM*^tdTomato^* neurons in sagittal sections of the spinal cord. Right image is an enlarged view of the boxed area. Scale bar (left), 500 μm. Scale bar (right), 50 μm. **(B)** Sorting of SOM*^tdTomato^* neurons in the spinal dorsal horn by flow cytometry. Spinal dorsal horn neurons from *SOM^Cre^* mice were used as controls (left). The P4 region represents Tomato-positive neurons, whereas the P5 region represents Tomato-negative neurons. **(C,D)** qPCR analysis of *Sst* **(C)** and *Cacna1h* **(D)** gene expression levels in Tomato-negative and Tomato-positive cells. *P < 0.05, **P < 0.01, Student’s unpaired *t*-test. FSC, forward scatter. SSC, side scatter.

Using this probe, we observed prominent localization of *Cacna1h* in the superficial laminae of the spinal dorsal horn (Figure 1B). As SOM^+^ neurons were deemed a key subpopulation of excitatory neurons in spinal dorsal horn mediating mechanical pain (Duan et al., 2014), we observed the localization of *Cacna1h* in SOM^+^ neurons. Staining of *in situ* hybridization in *SOM-Cre; ROSA26*^CAG–loxP–STOP–loxP–tdTomato^** mice showed prominent colocalization of *Cacna1h* and SOM*^tdTomato^* (Figure 1C). As previously described (Duan et al., 2014), SOM*^tdTomato^* cells were primarily localized in laminae II of the spinal cord, with scattered expression in superficial laminae I and deeper laminae III-IV (Figures 1C, 2A). Quantification analysis of the staining in laminae II showed that 82.8% of SOM*^tdTomato^* neurons expressed *Cacna1h* (Figure 1D). Conversely, 74.8% of *Cacna1h*-positive cells were SOM positive.

In contrast, sporadic localization of *Cacna1h* was observed in Dyn*^tdTomato^* neurons (Supplementary Figure 1A), which serves as a key subpopulation of inhibitory neurons in spinal dorsal horn to gate the transmission of Aβ pathway (Duan et al., 2014). The distribution of Dyn*^tdTomato^* neurons was concentrated in laminae I-II. Quantification analysis of the staining in laminae II showed that 31.0% of Dyn*^tdTomato^* neurons expressed *Cacna1h* (Supplementary Figure 1B). Conversely, 16.0% of *Cacna1h*-positive cells were localized in Dyn*^tdTomato^* neurons. Although most of the Dyn*^tdTomato^* neurons were inhibitory, excitatory properties of Dyn^+^ neurons in laminae I-II have been reported in previous studies (Huang et al., 2018). Therefore, the properties of Dyn*^tdTomato^* neurons exhibiting colocalization with *Cacna1h* remain to be determined. Taken together, these results indicated that *Cacna1h* is highly expressed in spinal SOM^+^ excitatory interneurons in laminae II.

Meanwhile, we observed the localization of *Cacna1g* and *Cacna1i* in the spinal dorsal horn using *in situ* hybridization. *Cacna1g* was distributed in both the superficial and deep dorsal horn (Supplementary Figure 2A). Colocalization of *Cacna1g* and SOM*^tdTomato^* could also be observed. In contrast, *Cacna1i* was localized in the deep dorsal horn (Supplementary Figure 2B).

### High Expression of *Cacna1h* in Spinal SOM^+^ Neurons

To further determine the abundance of *Cacna1h* in spinal SOM^+^ neurons, we collected spinal SOM*^tdTomato^* neurons using fluorescence-activated cell sorting (FACS). Acute dissociated spinal dorsal horn neurons from *SOM-Cre; ROSA26*^CAG–loxP–STOP–loxP–tdTomato^** mice were separated by FACS depending on the red fluorescence level (Figure 2B). *SOM^Cre^* mice were used as controls. As expected, *Sst* expression was significantly higher in the neuronal population of Tomato^+^ (SOM^+^) neurons than in Tomato^–^ (SOM^–^) cells (Figure 2C). Similarly, *Cacna1h* expression was higher in SOM^+^ neurons than in the SOM^–^ cell population (Figure 2D). Altogether, these results further demonstrated the enrichment of *Cacna1h* in spinal SOM^+^ neurons.

### Deficits in Light Touch and Acute Mechanical Pain After Selective Knockdown of *Cacna1h* in Spinal SOM^+^ Neurons

**FIGURE 3:**
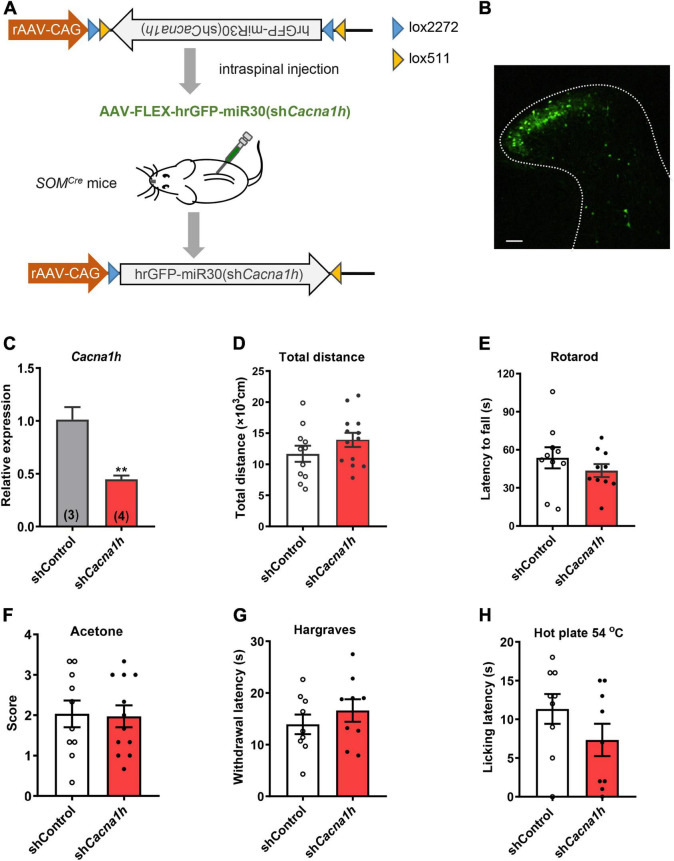
Knockdown of *Cacna1h* in spinal SOM^+^ neurons does not affect motor ability or thermal sensations in naïve mice. **(A)** Schematic illustration of viral-mediated knockdown of *Cacna1h* in spinal SOM^+^ neurons through intraspinal injection. Non-silence shRNA virus was used as a control. **(B)** Expression of the GFP-tagged virus in the spinal cord. Scale bar, 100 μm. **(C)** qPCR analysis of the relative expression level of *Cacna1h* 28 d after injection of *Cacna1h* knockdown virus. Non-silence shRNA virus was used as a control. **(D–H)** Behavioral tests of motor ability and thermal sensations in the mice after knockdown of *Cacna1h* in spinal SOM^+^ neurons. The total distance traveled in the open field **(D)**, the latency to fall in the rotarod **(E)**, the behavioral responses to acetone-evoked cooling **(F)**, the withdrawal latency in the Hargreaves test **(G)** and the licking latency in the hot plate at 54°C **(H)** were unaffected by *Cacna1h* knockdown in spinal SOM^+^ neurons. Student’s unpaired *t*-test.

To investigate the role of *Cacna1h* in spinal SOM^+^ neurons in tactile and pain transmission, we selectively knocked down *Cacna1h* in *SOM^Cre^* mice through intraspinal injection of Cre-dependent adeno-associated virus 9 (AAV9) carrying shRNA targeting *Cacna1h* (Figure 3A). Non-silence shRNA virus was used as a control. Twenty-eight days after virus injection, we observed that expression of the GFP-tagged virus was concentrated in the superficial laminae of the spinal dorsal horn (Figure 3B), while expression of the virus in the deep laminae and ventral horn was negligible. Viral expression was also limited on the ipsilateral side of the intraspinal injection (Supplementary Figure 3A). In addition, no GFP signal was detected in the DRG (Supplementary Figure 3B), excluding the possibility of knocking down *Cacna1h* in DRG. Efficient knockdown of *Cacna1h* in spinal dorsal horn was confirmed by qPCR analysis (Figure 3C).

First, we assessed motor ability and sensorimotor coordination using the open field test and rotarod test, respectively. No significant difference in total distance traveled in the open field test was observed (Figure 3D). The latency to fall in the rotarod was also not significantly different between the *Cacna1h* knockdown and control groups (Figure 3E). In addition, thermal sensation was assayed using the acetone test, Hargreaves apparatus and hot plate test. The cold sensitivity of the hind paw was not significantly different between the two groups (Figure 3F). The heat sensitivity of the hind paw based on the reflexive (Figure 3G) and non-reflexive (Figure 3H) responses also exhibited no significant difference. Therefore, knockdown of *Cacna1h* in spinal SOM^+^ neurons did not affect motor or thermal sensation under basal conditions.

**FIGURE 4:**
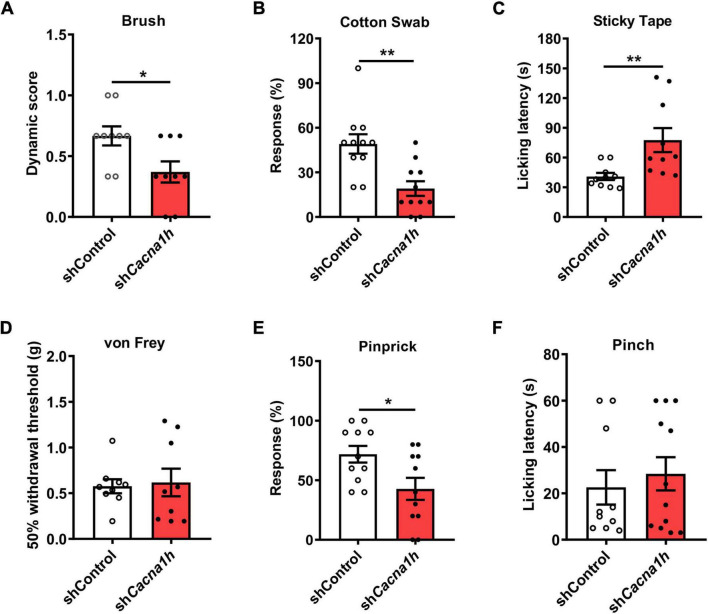
Knockdown of *Cacna1h* in spinal SOM^+^ neurons decreases the response to light touch and noxious mechanical stimulation in naïve mice. **(A–C)** Behavioral tests of the tactile sensation. Decreased the response to brush stimulation **(A)** and “pulled out” cotton swabs **(B)**, and increased licking latency to sticky tape **(C)** were observed after *Cacna1h* knockdown in spinal SOM^+^ neurons. **(D)** The 50% withdrawal threshold to von Frey filament stimulation was unaffected by *Cacna1h* knockdown in spinal SOM^+^ neurons. **(E)** The percentage of response to pinprick stimulation was reduced after *Cacna1h* knockdown in spinal SOM^+^ neurons. *P < 0.05, Student’s unpaired *t*-test. **(F)** The licking latency to pinch stimulation was not affected by *Cacna1h* knockdown in spinal SOM^+^ neurons. *P < 0.05, **P < 0.01, Student’s unpaired *t*-test.

Next, we performed a series of behavioral tests to assess tactile and acute pain sensations. In the brush assay, the plantar surface of the hind paw was stimulated by light stroking with a paintbrush, and the response of the mice was scored. *Cacna1h* knockdown mice exhibited reduced scores compared to the non-silence control group (Figure 4A). *Cacna1h* knockdown mice also displayed a reduced response to a “puffed out” cotton swab (Figure 4B). Additionally, *Cacna1h* knockdown mice showed a longer latency to biting or licking to remove the tape stuck onto the hind paw compared to the non-silence control (Figure 4C). These results indicate that knockdown of *Cacna1h* in spinal SOM^+^ neurons impairs light touch sensation in mice.

Furthermore, the threshold of mechanical pain and responsiveness to noxious mechanical stimuli in the hind paw were assayed. In the von Frey filament test, no significant difference in the 50% withdrawal threshold was detected after *Cacna1h* knockdown (Figure 4D). However, the percentage of response to pinprick stimulation was reduced after *Cacna1h* knockdown (Figure 4E). The licking latency to pinch stimulation was not significantly different between the two groups (Figure 4F). Altogether, these data indicate that knockdown of *Cacna1h* in spinal SOM^+^ neurons reduces the responses to noxious mechanical stimuli in mice.

### Attenuation of Heat Hyperalgesia and Mechanical Allodynia in Pathological Pain Following Selective Knockdown of *Cacna1h* in Spinal SOM^+^ Neurons

**FIGURE 5:**
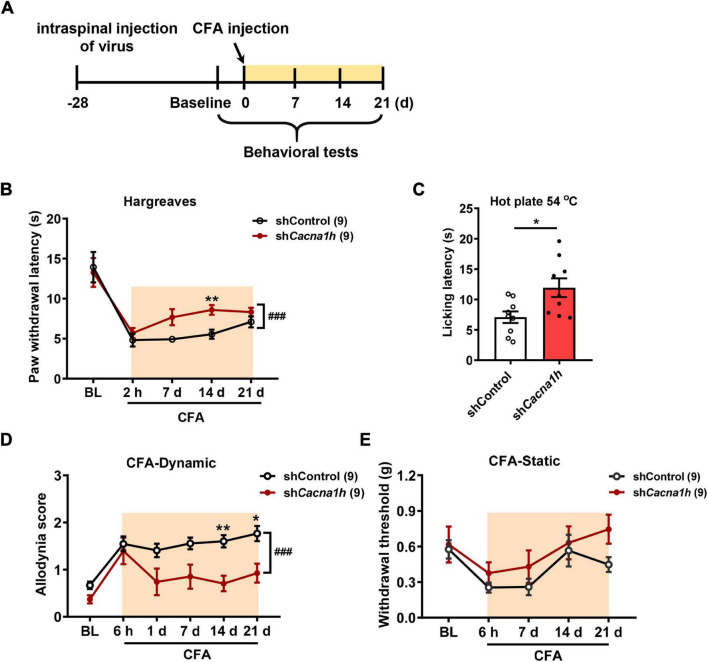
Knockdown of *Cacna1h* in spinal SOM^+^ neurons attenuated heat hyperalgesia and dynamic allodynia but not static allodynia in a CFA-induced chronic inflammatory pain model in mice. **(A)** Schematic illustration of the timeline of virus injection, model establishment and behavioral tests. Non-silence shRNA virus was used as a control. **(B)** The withdrawal latency in the Hargreaves test was increased after *Cacna1h* knockdown in spinal SOM^+^ neurons in the CFA model. ^###^ *P* < 0.001, two-way ANOVA with Sidak *post hoc* analysis. **P < 0.01, difference between the two groups at the corresponding time points. **(C)** Licking latency in the hot plate of 54°C was elongated by *Cacna1h* knockdown in spinal SOM^+^ neurons at 7 days post CFA injection. *P < 0.05, Student’s unpaired *t*-test. **(D,E)** The dynamic allodynia score was reduced **(D)**, but the static allodynia reflected as the 50% withdrawal threshold was not affected **(E)** by *Cacna1h* knockdown in spinal SOM^+^ neurons in the CFA model. ^###^ *P* < 0.001, two-way ANOVA with Sidak *post hoc* analysis. *P < 0.05, **P < 0.01, difference between the two groups at the corresponding time points. BL, baseline.

In subsequent studies, we examined the effects of *Cacna1h* knockdown in spinal SOM^+^ neurons on pain behaviors in chronic pain models. Twenty-eight days after virus injection, CFA was injected into the hind paw to establish an inflammatory pain model, and pain behaviors were evaluated at different time points (Figure 5A). The Hargreaves test demonstrated that the paw withdrawal latency was increased by *Cacna1h* knockdown (Figure 5B). The analgesic effect caused by *Cacna1h* knockdown was prominent on day 14 post CFA injection but diminished on day 21 post CFA injection. The hot plate test at 54°C also showed increased licking latency on day 7 post CFA injection in mice with *Cacna1h* knockdown (Figure 5C). In addition, we assayed the dynamic and static mechanical allodynia evoked by brush and von Frey filaments, respectively. *Cacna1h* knockdown significantly attenuated dynamic allodynia on day 14 and 21 post CFA injection (Figure 5D). However, the extent of static allodynia was not altered by *Cacna1h* knockdown (Figure 5E). Altogether, these data indicate that *Cacna1h* knockdown in spinal SOM^+^ neurons attenuates heat hyperalgesia and dynamic allodynia in chronic inflammatory pain.

**FIGURE 6:**
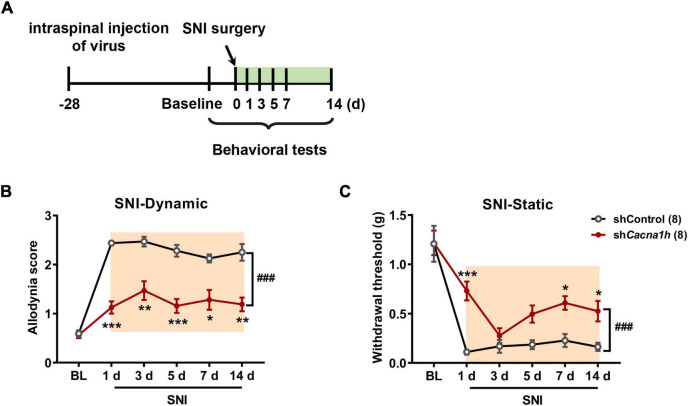
Knockdown of *Cacna1h* in spinal SOM^+^ neurons attenuated dynamic and static allodynia in an SNI-induced neuropathic pain model in mice. **(A)** Schematic illustration of the timeline of virus injection, model establishment and behavioral tests. Non-silence shRNA virus was used as a control. **(B,C)** Both the dynamic allodynia score **(B)** and the static allodynia reflected as the 50% withdrawal threshold **(C)** were reduced by *Cacna1h* knockdown in spinal SOM^+^ neurons in the SNI model. ^###^ *P* < 0.001, two-way ANOVA with Sidak *post hoc* analysis. *P < 0.05, **P < 0.01, ***P < 0.001, difference between the two groups at the corresponding time points. BL, baseline.

Similarly, we observed the impact of *Cacna1h* knockdown in spinal SOM^+^ neurons on pain behaviors in the SNI model of mice (Figure 6A). As withdrawal latency to the heat stimuli was unchanged in the SNI model (Decosterd and Woolf, 2000), we focused on mechanical allodynia in subsequent studies. *Cacna1h* knockdown significantly attenuated dynamic allodynia from days 1 to 14 postsurgery (Figure 6B). Static allodynia was also attenuated by *Cacna1h* knockdown on days 1, 7 and 14 postsurgery (Figure 6C). Therefore, *Cacna1h* knockdown in spinal SOM^+^ neurons attenuates both dynamic and static allodynia in chronic neuropathic pain.

### Nerve-Injury-Induced Aβ Inputs to I–IIo Neurons Is Attenuated Following Selective Knockdown of *Cacna1h* in Spinal SOM^+^ Neurons

**FIGURE 7:**
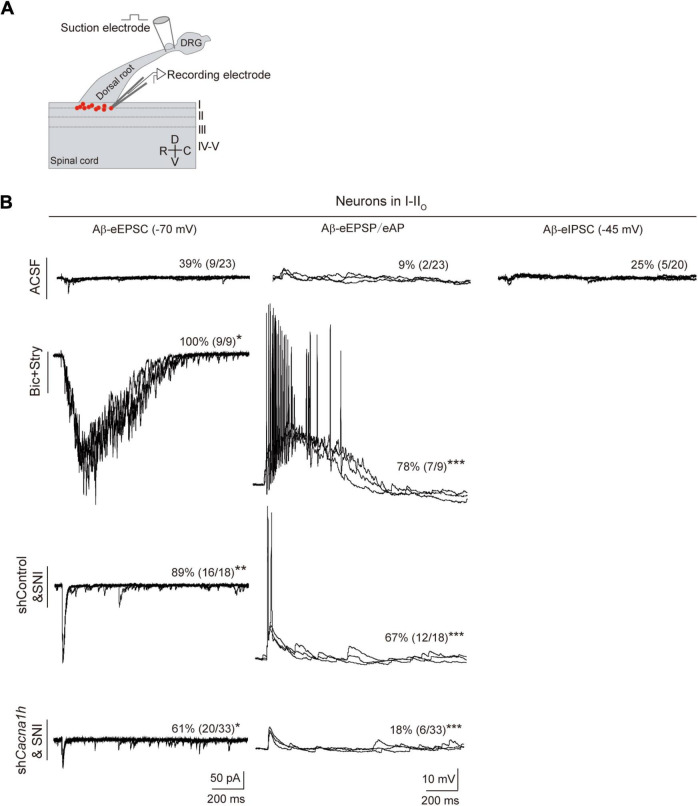
Knockdown of *Cacna1h* in spinal SOM^+^ neurons partially closed the superficial Aβ pathway in neuropathic pain. **(A)** Schematic drawing of patch clamp recordings on parasagittal spinal cord slice with dorsal root and DRG attached and recorded neurons (red dots). **(B)** Representative traces and percentage of neurons in laminae I-II_*o*_ with Aβ-eEPSC (left), Aβ-eAP (middle) and Aβ-eIPSC (right) in naïve mice under normal ACSF, ACSF containing bicuculline (Bic) plus strychnine (Stry), non-silence-shRNA-injected *SOM^Cre^* mice with SNI (shControl&SNI) and *Cacna1h*-shRNA-injected *SOM^Cre^* mice with SNI (sh*Cacna1h*&SNI). All data are represented as percentage. *P < 0.05. **P < 0.01. ***P < 0.001. Chi-square test.

In humans, Aβ fibers can sense touch and mediate allodynia. When mechanical hypersensitivity occurs, polysynaptic Aβ pathway from laminae III–V to laminae I–IIo neurons are revealed (Lu et al., 2013; Duan et al., 2014). We thus assessed whether Aβ pathway to superficial laminae is affected by knockdown of *Cacna1h* in spinal SOM^+^ neurons. Spinal cord slices with intact Aβ-inputs were prepared. Patch clamp recordings were then performed on neurons within laminae I-IIo (Figure 7A) where pain output neurons are mostly located (Wercberger and Basbaum, 2019). In naïve mice with ACSF, 39% (9 of 23) of neurons showed small Aβ-evoked excitatory postsynaptic currents (Aβ-eEPSCs) following dorsal root stimulation at 25 μA electric intensity which can specifically activate Aβ afferents (Duan et al., 2014). When the membrane potential was clamped at -45 mV, 25% (5 of 20) of neurons have detectable Aβ-evoked inhibitory postsynaptic currents (Aβ-eIPSCs), indicating concurrent feed-forward inhibition. Under current clamp recording, only 9% (2 of 23) of neurons fired APs (Figure 7B, “ACSF” group). Under disinhibition condition by bicuculline (10 μM) and strychnine (2 μM) to block inhibitory GABA_*A*_ and glycine receptors, respectively, 100% (9 of 9) of I-IIo neurons showed Aβ-eEPSCs and 78% (7 of 9) neurons fired APs, in comparison with 39% (*P* < 0.05) and 9% (*P* < 0.001) under normal ACSF (Figure 7B, “Bic + Stry” group), respectively, further proving the gated superficial Aβ pathway. Following SNI in shControl mice, the significant increase in the percentage of neurons of I-IIo displaying Aβ-eEPSCs and Aβ-eAPs were observed: from 39 to 89% (16 of 18, *P* < 0.01), and from 9 to 67% (18 of 32, *P* < 0.001) in naïve and SNI mice (Figure 7B, “shControl&SNI” group), respectively, indicating that nerve injury can open the gated superficial Aβ pathway. However, in *Cacna1h*-shRNA injected *SOM^Cre^* mice with SNI, we observed a decrease in the percentage of neurons with Aβ-eEPSCs and Aβ-eAPs: from 89 to 61% (20 of 33, *P* < 0.05), and from 67 to 18% (6 of 33, *P* < 0.001) (Figure 7B, “sh*Cacna1h*&SNI” group), respectively, relative to shControl group. Taken together, superficial Aβ-fiber inputs pathway could be one candidate substrate for attenuated mechanical hypersensitivity after selective knockdown of *Cacna1h* in spinal SOM^+^ neurons.

**FIGURE 8:**
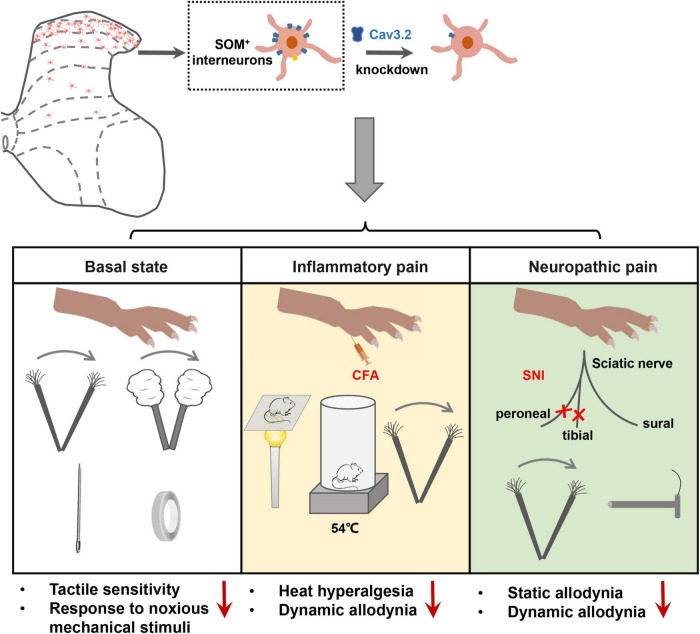
Cav3.2 channels in spinal SOM^+^ neurons are involved in the sensory processing of light touch in the basal state and contribute to heat hyperalgesia and mechanical allodynia in pathological pain, including inflammatory pain and neuropathic pain. In the current work, we demonstrated that the *Cacna1h* gene was enriched in SOM^+^ neurons in the spinal dorsal horn. After knockdown of *Cacna1h* expression in spinal SOM^+^ neurons by intraspinal injection of Cre-dependent virus, the mice displayed a lower response to light touch, including brush, cotton and tape stimulation, and showed a decreased response to the noxious mechanical stimulation in the basal state. In the pathological state, knockdown of *Cacna1 h* in spinal SOM^+^ neurons attenuated thermal hyperalgesia and dynamic mechanical allodynia behaviors in the inflammatory pain model and both dynamic and static mechanical allodynia behaviors in the neuropathic pain model.

## Discussion

In the current work, we investigated the localization and function of Cav3.2 in the spinal dorsal horn in mechanosensation and pain processing. The main findings are summarized as follows. First, a high expression level of Cav3.2 was observed in SOM^+^ neurons in spinal dorsal horn. Second, specific disruption of the expression of Cav3.2 in SOM^+^ neurons impaired light touch sensation and attenuated acute mechanical pain in naïve mice. Third, specific disruption of the expression of Cav3.2 in SOM^+^ neurons attenuated heat hyperalgesia and dynamic allodynia in inflammatory pain as well as dynamic and static allodynia in neuropathic pain. Fourth, Cav3.2 knockdown in spinal SOM^+^ neurons reduced the nerve-injury-induced Aβ inputs to I–IIo neurons. Altogether, our work reveals a functional role of Cav3.2 in tactile and pain processing at the level of the spinal cord in addition to its well-established peripheral role in pathological pain.

### T-Type Calcium Channels and Neuronal Excitability

T-type Ca^2+^ channels exhibit a typical window current, which contributes to the resting membrane potential (Dreyfus et al., 2010). In addition, the low-threshold property of activation makes this type of channels a major factor for the initial membrane depolarization before sodium spikes (Perez-Reyes, 2003). T-type Ca^2+^ channels are also involved in shaping the action potentials. During the repolarization phase, they are activated and contribute to a hump in the falling phase, leading to action potential broadening in the projection neurons in spinal laminae I (Ikeda et al., 2003; Cain and Snutch, 2010). Moreover, activation of only a small fraction of T-type Ca^2+^ channels is required to generate robust high-frequency burst firing of neurons (Dreyfus et al., 2010). Altogether, T-type Ca^2+^ channels regulate neuronal subthreshold excitability as well as action potential firing.

As a major subtype of T-type Ca^2+^ channels, Cav3.2 channel was reported to be involved in the multiple controls of neuronal excitability in laminae II (Candelas et al., 2019). Cav3.2 deletion led to remodeling of the kinetics of the action potentials, including alteration of the half-width, time-to-peak, threshold potential and peak potential. Overall, Cav3.2 deletion reduced the neuronal proportions of transient firing, rebound depolarization, and action potential pairing. Here, we analyzed the effect of Cav3.2 knockdown on the neurotransmission from Aβ fibers to spinal superficial neurons. In accordance with the previous studies (Duan et al., 2014; Cheng et al., 2017), our work proved the opening of Aβ inputs to superficial spinal neurons in neuropathic pain. Importantly, this superficial Aβ pathway was partially closed by Cav3.2 knockdown in SOM^+^ neurons.

### Expression of T-Type Calcium Channels in the Spinal Dorsal Horn

An early study of *in situ* hybridization demonstrated the expression of T-type Ca^2+^ channels in the spinal cord (Talley et al., 1999). Later, functional evidence of the expression of T-type Ca^2+^ channels in the spinal dorsal horn was provided. T-type Ca^2+^ channels were expressed in approximately 80% spinal laminae I neurons (Harding et al., 2021). Moreover, they participate in activity- and calcium-dependent long-term potentiation at synapses between nociceptive afferents and laminae I projection neurons, which mediate the development of pain hypersensitivity (Ikeda et al., 2003). Recently, it was reported that the T-current could be recorded in approximately 45% of spinal laminae II (substantia gelatinosa) neurons (Wu et al., 2018). Further morphological analysis indicated that most of the neurons expressing T-currents are islet neurons, which are thought of as inhibitory neurons. Altogether, previous studies showed that T-type Ca^2+^ channels were expressed in both projection neurons and inhibitory interneurons in spinal dorsal horn.

With respect to the subtypes of T-type Ca^2+^ channels in spinal dorsal horn, the previous electrophysiological studies provided the key evidence (Francois et al., 2015; Bernal Sierra et al., 2017). Selective deletion of Cav3.2 in the spinal dorsal horn reduced the number of neurons exhibiting single spiking, transient and irregular tonic patterns (Candelas et al., 2019). In contrast, deletion of Cav3.1 reduced the proportion of neurons that exhibited a regular tonic firing pattern. Combined with our findings, we reasoned that Cav3.2 might be the major subtype of T-type Ca^2+^ channels in spinal excitatory interneurons, while Cav3.1 might be the predominant subtype of T-type Ca^2+^ channels in inhibitory interneurons.

### High Expression of Cav3.2 in SOM^+^ Neurons in the Spinal Dorsal Horn

Our experiments revealed that approximately three-quarters (74.8%) of Cav3.2-expressing cells were SOM^+^ neurons, while 16.0% were Dyn^+^ neurons in spinal laminae II. This finding is close to the data reported by Candelas et al. (2019). They showed that 70.8% of GFP-Cav3.2 was localized in Tlx3^+^ excitatory neurons and that 13.1% was localized in Pax2^+^ inhibitory neurons. Therefore, SOM^+^ interneurons might constitute the majority population of Cav3.2-expressing excitatory neurons.

Meanwhile, we found that 82.8% of SOM^+^ neurons expressed Cav3.2. As SOM^+^ interneurons represent 37% of total neurons in laminae II (Duan et al., 2014), we can infer that 30.6% (37 × 82.80%) of neurons in laminae II are Cav3.2-positive. In addition, as excitatory interneurons account for three-quarters of neurons in laminae II in the mouse dorsal horn (Peirs et al., 2020), we can also infer that Cav3.2 is expressed in 40.8% (30.6/75%) of excitatory interneurons in laminae II, which constitute the intermediate for the transmission of Aβ input to the superficial dorsal horn.

Previous transcriptomic data in mouse spinal dorsal horn showed that *Cacna1h* was abundant in excitatory DE-1–2, 5–7 and inhibitory DI-3 clusters (D, dorsal; E, excitatory; I, inhibitory) (Sathyamurthy et al., 2018). Among them, DE-2, 5 and 7 clusters coexpress *Sst* and *Cacna1h*. In another transcriptomic study, *Cacna1h* was found to be enriched in excitatory Glut4, 6–8 and 10 and inhibitory Gaba1 clusters of spinal neurons. Coexpression of *Sst* and *Cacna1h* was found in clusters Glut4, 6 and 7 (Haring et al., 2018). Combined with our findings, coexpression of *Sst* and *Cacna1h* exists mainly in the excitatory neurons in the spinal dorsal horn. However, expression of *Cacna1h* in inhibitory neurons could not be excluded.

Spinal dorsal horn neurons transiently expressing vesicular glutamate transporter 3 (tVGLUT3) have been shown to mediate dynamic allodynia but not static allodynia behaviors (Cheng et al., 2017). As partial overlap between transient VGLUT3-positive neurons and SOM^+^ neurons exists in the spinal dorsal horn, this subpopulation might mediate the effects of Cav3.2 knockdown in SOM^+^ neurons in dynamic allodynia.

### The Heterogeneous Properties of Cav3.2-Expressing Neurons in the Spinal Cord

Previous studies have indicated that approximately 40% of Cav3.2-expressing neurons are calbindin positive and PKCγ positive (PKCγ^+^) (Candelas et al., 2019). In reverse, approximately 90% of PKCγ^+^ neurons are Cav3.2-positive. PKCγ-expressing neurons have been proposed to be a key population leading to mechanical allodynia in neuropathic pain (Lu et al., 2013; Petitjean et al., 2015). Meanwhile, colocalization of SOM^+^ and PKCγ^+^ neurons has been demonstrated (Duan et al., 2018). Hence, the anti-allodynic effects of *Cacna1h* knockdown in the SNI model might be attributed to disruption of the function of the PKCγ^+^ subpopulation. In addition, 20% of Cav3.2-expressing cells are calretinin-positive (CR^+^) cells, which are thought to specifically mediate mechanical allodynia in inflammatory pain (Peirs et al., 2021). Therefore, disruption of the function of the CR^+^ subpopulation in Cav3.2-expressing neurons might contribute to the anti-allodynic effects of *Cacna1h* knockdown in the CFA model as observed here.

### The Peripheral and Central Roles of Cav3.2 in Pain Hypersensitivity

The function of Cav3.2 in peripheral sensory neurons has been well established. Cav3.2 is the major subtype of T-type Ca^2+^ channels in the DRG (Bourinet et al., 2005; Rose et al., 2013). It is expressed in major populations of mechanoreceptors, including Aδ-LTMRs and C-LTMRs (Francois et al., 2015). Peripheral Cav3.2 was demonstrated to participate in neuropathic pain induced by the SNI model (Francois et al., 2015; Kang et al., 2018), spinal verve ligation model (Lai et al., 2017; Gomez et al., 2020), chronic constriction injury (Bourinet et al., 2005; Jagodic et al., 2008), diabetes mellitus (Latham et al., 2009; Messinger et al., 2009; Obradovic et al., 2014), chemotherapeutic agents (Okubo et al., 2011; Li et al., 2017), chronic visceral pain caused by irritable bowel syndrome (Cain and Snutch, 2013), postsurgical pain (Joksimovic et al., 2018) and insulin-like growth factor 1-induced hyperalgesia (Zhang et al., 2014). Upregulated Cav3.2 expression or increased T-current in injured or uninjured DRG neurons has been demonstrated in pathological pain (Li et al., 2017; Chen et al., 2018; Kang et al., 2018; Shin et al., 2020). Downregulation of the expression of Cav3.2 through knockdown of the deubiquitinating enzyme USP5 or uncoupling USP5 from native Cav3.2 channels *via* intrathecal delivery of Tat peptide has been shown to relieve pain in both inflammatory pain and neuropathic pain models (Garcia-Caballero et al., 2014, 2016; Stemkowski et al., 2017; Joksimovic et al., 2018).

In contrast, the role of spinal Cav3.2 in pathological pain is poorly understood. A recent work reported that partial sciatic nerve ligation (PSNL) triggered an increase in both the mRNA and protein levels of Cav3.2 but not Cav3.1 or Cav3.3 in spinal dorsal horn (Feng et al., 2019). Moreover, both the proportion of T-current-expressing neurons and T current density in individual neurons were elevated in spinal laminae II neurons from PSNL rats, which could not be recapitulated in Cav3.2 knockout mice. In accordance with the above findings, our work provided direct evidence for functional role of Cav3.2 in spinal dorsal horn in pain hypersensitivity.

However, due to the lack of a specific blocker of Cav3.2, broad spectrum T-type calcium channels blockers are used for alleviating pain symptoms (Bourinet et al., 2005; Francois et al., 2015). For example, both the traditional anti-epileptic drug ethosuximide and the first-in-class CNS-penetrant drug Z944 have been shown to block T-type calcium channels and attenuate pain hypersensitivity (Zamponi, 2016; Snutch and Zamponi, 2018). Moreover, Z944 has been shown to alleviate pain at both the peripheral and spinal cord levels (Harding et al., 2021). 5bk, a novel T-type modulator, has also been used for the treatment of pain hypersensitivity in rodent pain models (Sekiguchi et al., 2016). However, ABT-639, a peripherally acting T-type calcium channel blocker, failed in clinical trials in patients with diabetic neuropathic pain (Ziegler et al., 2015). Therefore, CNS-penetrant T type calcium channel blockers or highly selective blockers of Cav3.2, such as the agent targeting its intracellular C-terminus (Wang et al., 2015), might be preferred for the development of analgesic agent.

In summary, our work has indicated that Cav3.2 in spinal SOM^+^ neurons serves as a critical molecule in mechanical pain. This finding revealed a molecular mechanism for spinal SOM^+^ neurons in mechanical pain and provided the hint for the potential analgesic effect of T-type Ca^2+^ channel blockers at the level of spinal cord.

## Data Availability Statement

The raw data supporting the conclusions of this article will be made available by the authors, without undue reservation.

## Ethics Statement

The animal study was reviewed and approved by Peking University Animal Care and Use Committee.

## Author Contributions

Y-RZ and YiZ designed the experiment. YiZ, Y-RZ, and JL performed the morphological studies. Y-RZ and LS performed the FACS studies. Y-RZ, FC, S-WG, H-NZ, and J-YJ performed the behavioral studies. YaZ and X-JS performed the electrophysiological recording. FC, Y-RZ, YW, YaZ, and YiZ analyzed the data and wrote the manuscript. All authors contributed to the article and approved the submitted version.

## Conflict of Interest

The authors declare that the research was conducted in the absence of any commercial or financial relationships that could be construed as a potential conflict of interest.

## Publisher’s Note

All claims expressed in this article are solely those of the authors and do not necessarily represent those of their affiliated organizations, or those of the publisher, the editors and the reviewers. Any product that may be evaluated in this article, or claim that may be made by its manufacturer, is not guaranteed or endorsed by the publisher.
