# Supplementary material for: The T-Type Calcium Channel Cav3.2 in Somatostatin Interneurons in Spinal Dorsal Horn Participates in Mechanosensation and Mechanical Allodynia in Mice
Source: Front Cell Neurosci. 2022 Apr 8;16:875726. doi: 10.3389/fncel.2022.875726 (PMC9024096; doi:10.3389/fncel.2022.875726)
Supplement: Supplementary file 1 [file Data_Sheet_1.DOCX]

# Supplementary Material

**Tissue clearing and imaging**

The lumbar segment of the spinal cord was harvested and fixed in 4% PFA at 4 °C for overnight. Samples were washed in 1 × PBS for 3 times and incubated in X-CLARITY™ hydrogel-initiator mixture solution at 4 °C for 24 h. Samples were polymerized in a vacuum of −90 kPa at 37 °C for 3 h and washed in 1 × PBS 3 times for 5 min each. The tissue-hydrogel hybrids were subsequently cleared in an SDS-based solution by the X-CLARITY™ electrophoretic tissue clearing system (Logos Biosystems, Annandale, VA, USA) at 37 °C for 20 h with 1.5-A current and 50 rpm pump speed. Cleared samples were washed in 1 × PBS for 3 times and incubated in X-CLARITY™ mounting solution for refractive index (RI) matching at 37 °C overnight. Finally, the images were imaged by Leica TCS SP8 two-photon microscopy with an APO 25× / NA 0.95 water lens (Leica, Germany).

# Supplementary Figures


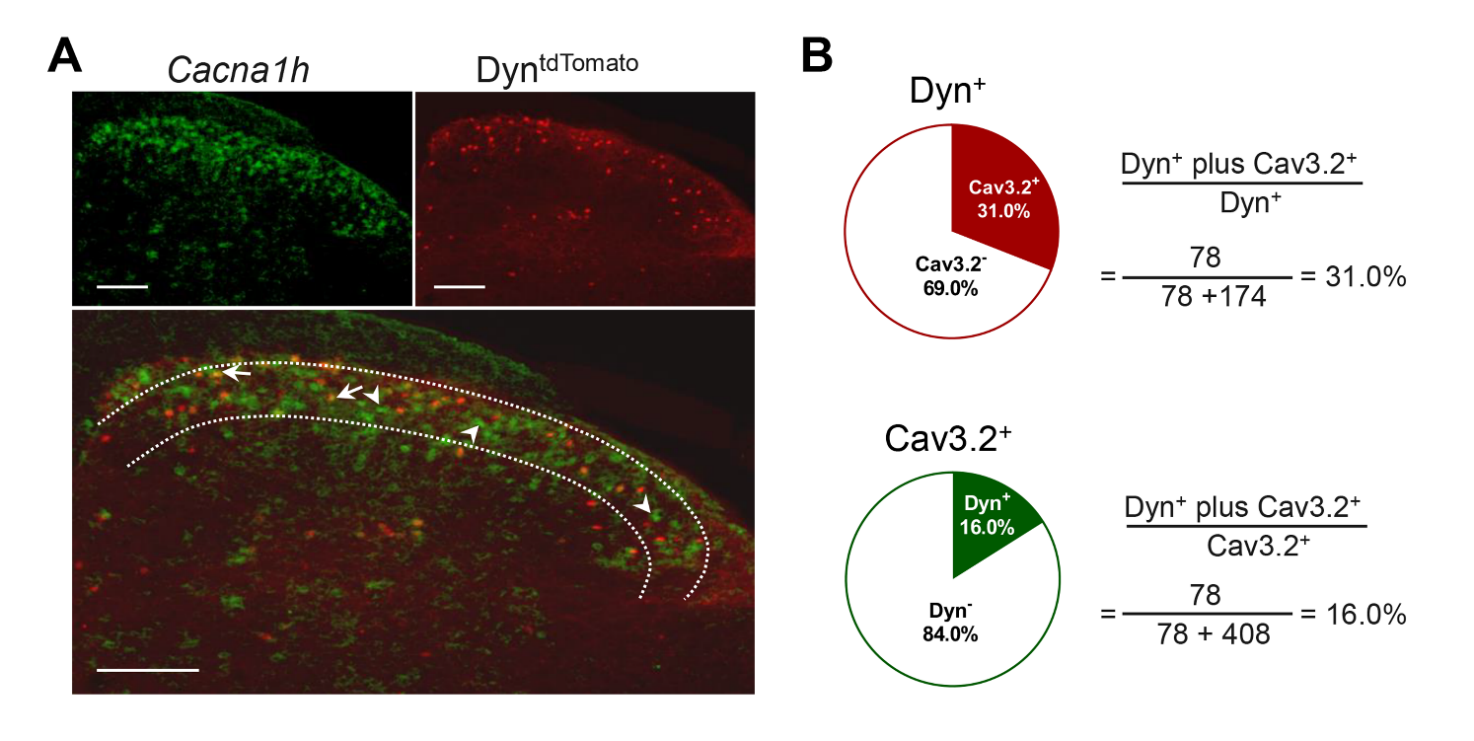


**Supplementary Figure 1.** Expression of *Cacna1h* in Dyn^+^ neurons in the spinal dorsal horn. (A) *In situ* hybridization staining of *Cacna1h* (left) and Dyn^tdTomato^ neurons (right) in the spinal dorsal horn. Superimposition of the images is shown below. Arrows indicate *Cacna1h* and Dyn^tdTomato^ double-positive cells, and arrowheads indicate *Cacna1h*-positive cells*.* (B) Quantification analysis of the percentage of double-positive cells in Dyn^+^ cells (top) and Cav3.2^+^ cells (below). Twelve hemisections of the spinal cord from 4 mice in each group were counted. Scale bar, 100 μm.


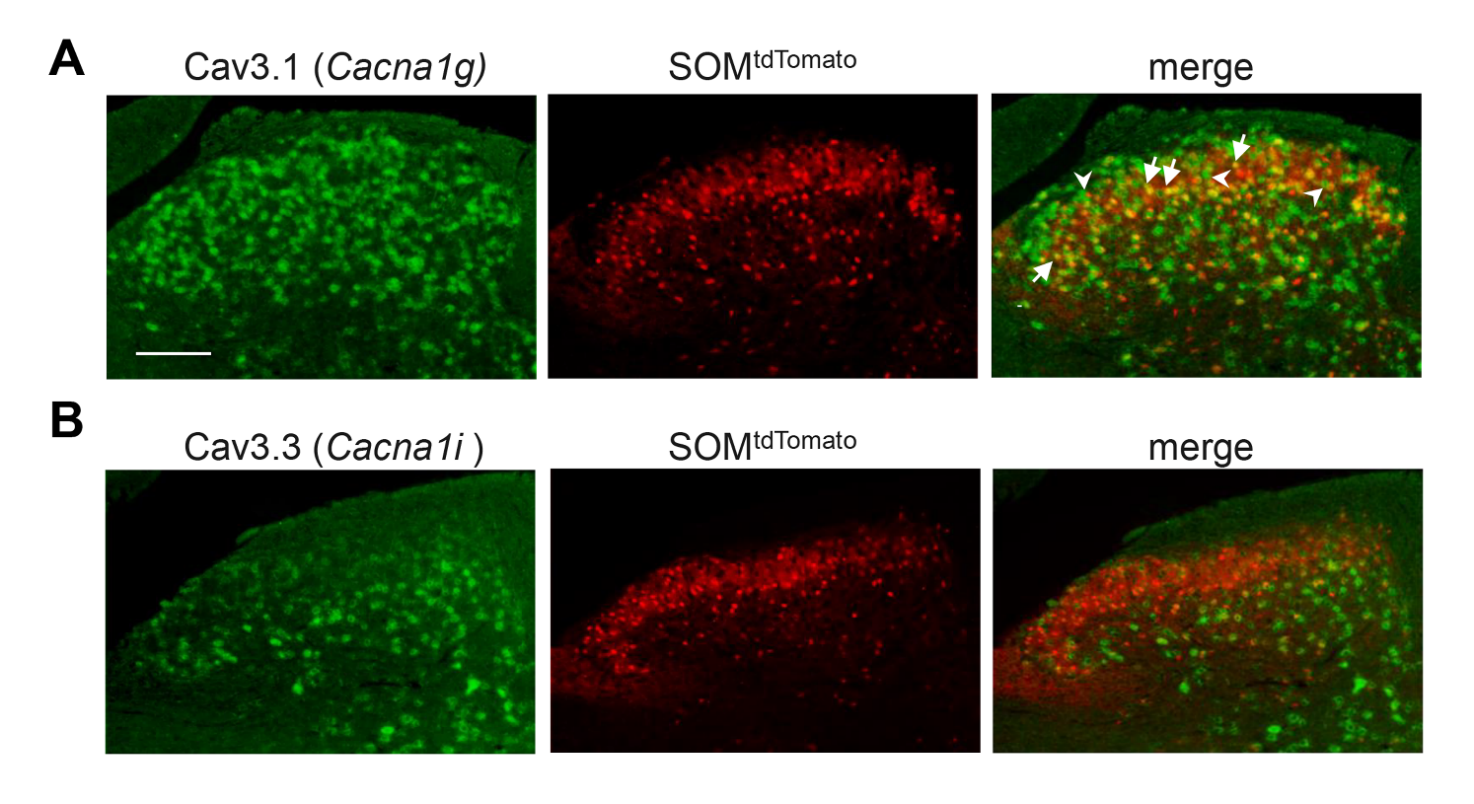


**Supplementary Figure 2.** Expression of Cav3.1/Cav3.3 in SOM^tdTomato^ neurons. *In situ* hybridization staining of *Cacna1g* (A, left) and *Cacna1i* (B, left) and SOM^tdTomato^ neurons (middle) in the spinal dorsal horn. Superimposition of the images is shown in the right. Arrows indicate *Cacna1g* and SOM^tdTomato^ double-positive cells, and arrowheads indicate *Cacna1g*-positive cells. Scale bar, 100 μm.


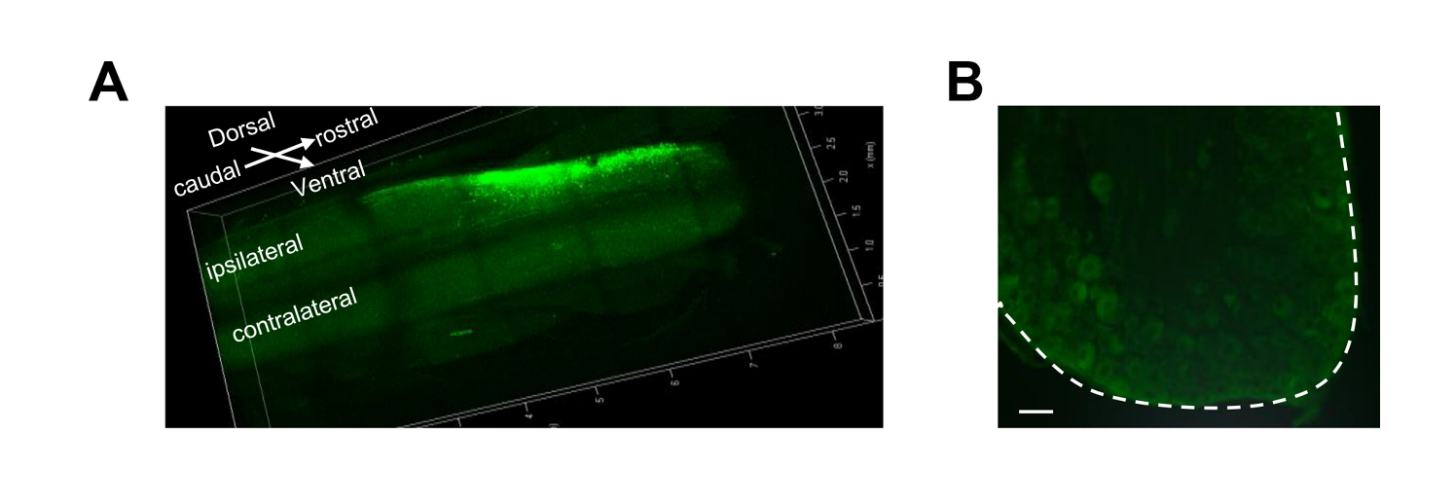


**Supplementary Figure 3.** Expression of *Cacna1h* knockdown virus in the spinal cord after intraspinal injection. (A) Representative image showing the unilateral expression of the virus in the spinal dorsal horn. The spinal cord was cleared using the CLARITY method and observed with two-photon microscopy. (B) No GFP expression in the DRG after intraspinal injection of virus. Scale bar, 100 μm.
